# Beyond Gonarthrosis in the Elderly: A Case Report of Subchondral Insufficiency Fracture of the Knee

**DOI:** 10.7759/cureus.34366

**Published:** 2023-01-30

**Authors:** Mariana Martins, Raquel Araújo, Rosana Pinheiro, Ana Costa, José Luís Carvalho

**Affiliations:** 1 Physical Medicine and Rehabilitation, Centro Hospitalar Universitário de Lisboa Central, Lisboa, PRT; 2 Physical Medicine and Rehabilitation, Centro Hospitalar Universitário do Porto, Porto, PRT; 3 Orthopaedic Surgery, Unidade Local de Saúde do Nordeste, Bragança, PRT; 4 Centro de Reabilitação do Norte, Centro Hospitalar de Vila Nova de Gaia/Espinho, Vila Nova de Gaia, PRT; 5 Intervention and Musculoskeletal Rehabilitation Unit, Centro de Reabilitação do Norte, Vila Nova de Gaia, PRT

**Keywords:** subchondral insufficiency fracture of the knee, orthopedic surgery, radiofrequency, rehabilitation, subchondral insufficiency fractures

## Abstract

Subchondral insufficiency fracture of the knee (SIFK) is a non-traumatic condition that has been, historically, associated with the elderly. Early diagnosis and management are essential to prevent evolution to subchondral collapse and secondary osteonecrosis, developing prolonged pain and functional losses.

This article presents the case of an 83-year-old patient with severe right knee pain with 15 months of evolution, with sudden onset, and no history of trauma or sprain. Upon observation, the patient presented with a limping gait, antalgic posture with the knee in semi-flexion, pain on palpation of the joint medial line, severe pain on passive mobilization, limited joint range of motion, and a positive McMurray test. The X-ray only demonstrated a gonarthrosis grade 1 in the Kellgren and Lawrence classification with medial compartment affection. Due to the exuberant clinical picture with marked functional compromise, as well as clinical radiological dissociation, MRI was requested to rule out SIFK, which was later confirmed. The therapeutic orientation was then adjusted with an indication for non-weight bearing and analgesia, as well as orientation to an orthopedics consultation to request a surgical evaluation.

SIFK is difficult to diagnose and may have an unpredictable outcome due to delayed treatment approaches. This clinical case encourages clinicians to consider subchondral fracture in the differential diagnosis of knee pain when an older patient, with subnormal radiographic findings, reports severe knee pain in the absence of overt traumatic injury.

## Introduction

Subchondral insufficiency fracture of the knee (SIFK) is a non-traumatic condition that occurs immediately below the cartilage of a joint and can be a potentially devastating disorder that may progress rapidly to severe osteoarthritis with articular surface collapse [1,2]. This entity has been associated with the elderly and osteoporotic women [1,2].

Many middle-aged adults experience disabling knee pain that can have a variety of etiologies [3]. One of the most frequent sources of disabling knee pain in persons over 60 years of age is symptomatic knee osteoarthritis [3]. However, nowadays, with the increasing number of older people, there is an ever-increasing need for accurate assessment of knee pain in this population [4]. Plain radiographs are typically used as initial imaging to investigate knee pain in middle-aged and older patients [3]. Therefore, MRI should be considered for older people presenting with sudden-onset non-traumatic knee pain and normal radiographs to ensure that other less common diagnoses do not fail [4-6].

The outcome of subchondral insufficiency fractures depends on several factors such as early fracture size, patient body mass index with a positive correlation with obesity, the severity of osteopenia, early diagnosis, and initial management [2,5]. These factors can influence the evolution of the lesion that can develop into osteonecrosis and osteochondral collapse, requiring surgical management, which makes us reflect on the importance of recognizing this entity to allow a correct therapeutic orientation, thus preventing a devastating outcome that could culminate in a long-term functional commitment [2-4].

## Case presentation

An 83-year-old woman with a known medical history of non-insulin-treated diabetes mellitus, hypertension, dyslipidemia, and obesity presented at the physical rehabilitation department, interventional physiatry consultation, for evaluation of right knee pain. The patient reported severe right knee pain, 8 in 10 on the Visual Analog Scale (VAS), with 15 months of evolution, with sudden onset, and no history of trauma or sprain. The pain significantly worsened with weight bearing. Due to the difficulty in gait and functional limitation, the started walking with one crutch and required moderate help with activities of daily living.

Upon observation, the patient presented a limping gait needing help from a third person. She presented with antalgic posture with the knee in semi-flexion, pain on palpation of the medial joint interline, and severe pain on passive mobilization, which was associated with limited joint range of motion (ROM) with 5° deficit on extension and 100° of flexion. In addition, she presented with a positive McMurray test to the medial compartment. All specific tests were doubtful due to the difficulty in evaluation because of pain inhibition. There was no swelling, redness, or other inflammatory signs in the right knee.

**Figure 1:**
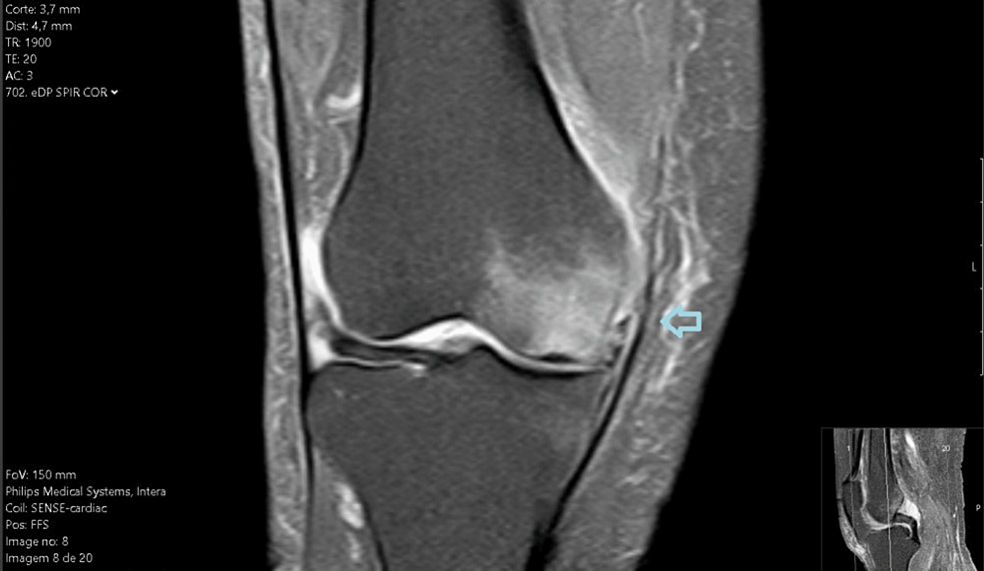
MRI of the right knee in the coronal plane presenting osteochondral lesion of the internal femoral condyle.

**Figure 2:**
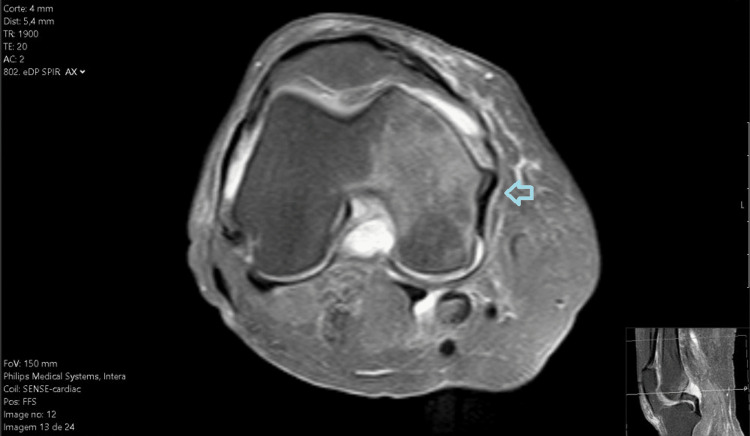
MRI of the right knee in the axial plane.

The X-ray demonstrated a gonarthrosis grade 1 in the Kellgren and Lawrence classification with medial compartment affection. Taking into account the early stage of degeneration, intra-articular administration with hyaluronic acid was attempted. Despite that, due to the exuberant clinical picture with marked functional compromise, clinical radiological dissociation, and the medical history and the age of the patient, MRI was requested to rule out SIFK. The MRI confirmed the diagnostic suspicion of an osteochondral lesion measuring approximately 13 × 13 × 4 mm of greater anteroposterior, transversal, and thickness axes, on the weight-bearing surface of the internal femoral condyle, without signs of instability, without apparently detached bone fragments, associated with marked bone medullary edema of the internal femoral condyle, and of a very slight degree in the medial peripheral aspect of the homolateral tibial plate (Figures 1, 2).

In this context, non-weight bearing and adaptation to a rehabilitation program were recommended, including gait training underwater with immersion level up to the axillary region. Clinic re-evaluation with a new MRI in four months was suggested.

**Figure 3:**
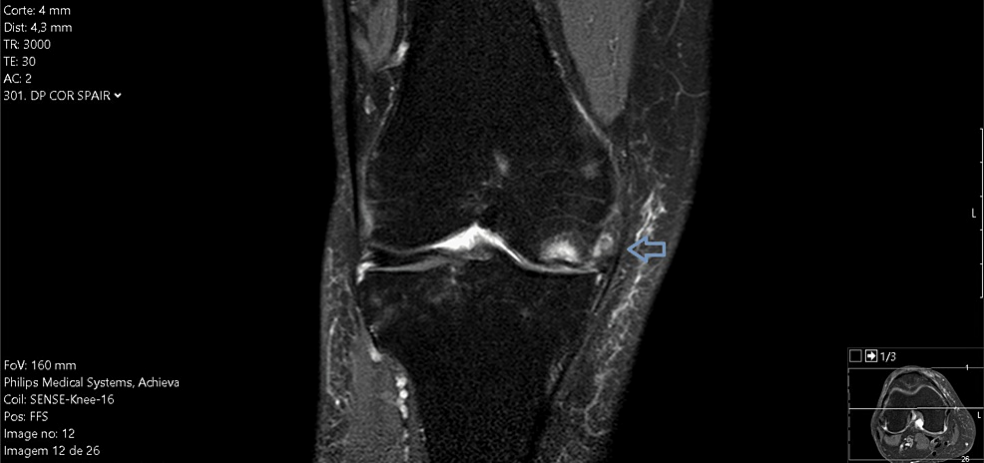
MRI of the right knee in the coronal plane (re-evaluation).

**Figure 4:**
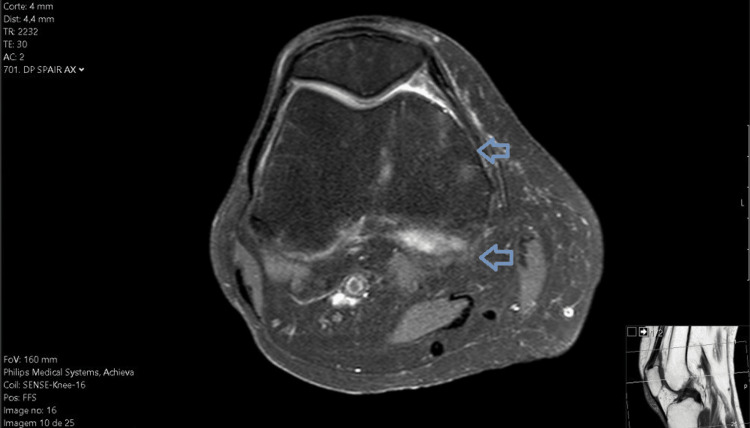
MRI of the right knee in the axial plane (re-evaluation).

The patient showed progressively improved knee joint mobility, maintaining functional limitation with the need for gait assistance. During the reassessment consultation, the patient still reported pain, although of slightly better (light/moderate) intensity. The MRI showed discrete evolution of the lesion with loss of complete cartilaginous thickness of the bearing surface of the internal femoral condyle with associated slight subchondral collapse, in the context of an osteochondral lesion due to grade IV chondropathy with subchondral fracture, probably due to insufficiency and without a detached osteochondral fragment or signs of instability (Figures 3, 4).

Taking into account the evolution, the patient was referred for an orthopedics consultation for evaluation of the surgical condition. After the evaluation, if there is no need for surgery, sensory denervation of the medial compartment of the knee, which includes an infrapatellar branch of the saphenous nerve and medial geniculate branches (superomedial and inferomedial) by thermal radiofrequency, would be considered.

## Discussion

SIFK is a microfracture allied to the inability of the bone to support normal and physiological loads, more frequently seen in middle-aged and older people [4]. Low bone mineral density and menopause may play a role in SIFK [4,5]. It has been described that SIFK predominantly involves the weight-bearing medial compartment of the knee and is often associated with medial meniscal tears [7-9]. Wilmot et al. described that meniscal extrusion actually may predispose to disease progression [8].

MRI plays an important role in the diagnosis of SIFK, which often goes unnoticed on initial radiographic examinations. These fractures can be recognized by the presence of a linear subchondral T1-T2 hypointense defect usually located underneath the articular cartilage on MRI with surrounding marrow and soft-tissue edema [9-12]. The radiological description follows the Koshino classification that contemplates four grades of evolution and is still often used as a reference for determining treatment strategies. Grade 1 is associated with edema-like marrow signal intensity/contusion without fracture line. Grade 2 is associated with subchondral fracture without cystic or osteonecrotic changes. Grade 3 is associated with subchondral fracture accompanied by cystic changes matching fluid-signal intensity. Grade 4 is associated with a subchondral fracture with subchondral collapse and overlying articular surface step-off [4,9,10]. Low-grade SIFK has a favorable outcome, typically resolving over time, whereas high-grade SIFK correlates with articular surface collapse, with evolution to fragmentation and osteonecrosis [2,9,11,12].

The differential diagnosis of subchondral insufficiency fractures includes spontaneous osteonecrosis of the knee (SONK) which is due to circulatory impairment, leading to the ischemic death of the cellular components of the bone and marrow [2,6]. Nowadays, it is widely accepted that SONK may correspond to distinct evolutive phases of the same disease mechanism. Nevertheless, due to their similar presentation and imaging findings, the relationship and distinction between SIFK and SONK remain unclear and a topic of debate in the literature [4,12]. The differential diagnosis of subchondral insufficiency fractures also includes osteoarthritis, being among the most frequent pathologies in this age group. This distinction is extremely important mainly due to the difference in therapeutic approaches between these two entities, controlled and supervised weight-bearing exercises being recommended for knee osteoarthritis, compared to the reduced weight bearing in SIFK to allow fracture healing [12,13].

The prognosis may range from full recovery to the rapid development of joint destruction [2]. For a successful recovery, prompt diagnosis followed by immediate non-weight bearing is crucial [2]. The use of crutches, non-steroidal anti-inflammatory agents, and cold therapy has been described as essential measures in conservative management [8-10]. The evolution in weight bearing should be according to the patient’s tolerance.

Disease progression has been related to initial gravity and continued weight bearing. [9] Baseline arthritis, older age, location of the insufficiency fracture on both the medial femoral condyle and medial tibial plateau, meniscal extrusion, and varus malalignment have been associated with progression to arthroplasty [6,14,15]. Approximately one-third of patients progressed to total knee arthroplasty [14].

The recovery period can be extremely prolonged. Recovery time of up to three years has been documented, with small progressive recovery gains; however, pain can become chronic and quite limiting in the long term [2]. Consequently, chronicity of the pain may become an extremely relevant factor to be taken into account in the therapeutic approach.

Given the reported success of nerve ablations in the treatment of chronic pain, Broida et al. have suggested that radiofrequency ablation (RFA) of the genicular nerve may be a viable minimally invasive treatment option for the management of patients with symptomatic SIFK and no surgical indication [10]. Genicular nerve RFA is a minimally invasive procedure to treat patients with intractable knee pain and is mostly applied in patients with symptomatic knee osteoarthritis recalcitrant to conservative modalities, those with persistent pain after knee replacement, those not accepted for surgical intervention due to medical comorbidities [16,17]. The sensory innervation of the knee is relayed through the superior lateral, superior medial, inferior medial, and inferior lateral genicular nerves, recurrent fibular nerve, and infrapatellar branch of the saphenous nerve [16,17]. The RFA involves sensitive denervation of the joint capsule through targeted delivery of radiofrequency energy to the genicular nerves, causing tissue heating and neural denaturation, thereby decreasing nociceptive signaling, and can be guided under ultrasonography or fluoroscopy [17]. To our knowledge, there is only one reported case of pain control with RFA in symptomatic SIFK [10].

## Conclusions

We present this clinical case to draw attention to this less frequent diagnosis, which can easily go unnoticed, especially at older ages. When there is a striking clinical and radiological dissociation, and after taking into account the patient’s age, gender, and medical history, we should suspect that a less common etiology may exist and request a directed MRI. A correct diagnosis is crucial, considering the disparity in the treatment of SFIK or pure osteoarthritis, which can interfere with the evolution and prognosis.
